# Carabids species diversity in Mediterranean beech forests

**DOI:** 10.3897/BDJ.10.e78291

**Published:** 2022-01-28

**Authors:** Roberto Pizzolotto

**Affiliations:** 1 Università della Calabria, Rende (CS), Italy Università della Calabria Rende (CS) Italy

**Keywords:** carabidae, Darwin Core terms, beech forests, pitfall traps, species activity density

## Abstract

**Background:**

Carabid beetles are gaining more and more attention in applied studies on environmental monitoring or evaluation of natural resources, probably because they can be used as model organisms. Data on the biology and species richness and abundance of carabids could give valuable information in such applied studies, but online resources are not so informative, at least for Italy. To start filling this gap, a data table (Darwin Core formatted) was uploaded in GBIF database. The table is the result of a pitfall trapping survey of carabids living in a small part of the beech forest ecosystem of the Calabria Region (Italy). Amongst the collected species, few were very abundant, which are likely to be the characterising species of the studied ecosystem.

**New information:**

Online datasets of Italian carabids are absent and information about the species biology, richness and abundance often lies in hard-copy papers. The dataset provided here is the first tentative approach, at least for the Italian fauna, to propose a formal structure for data on carabids acquired by field surveys and to give open access to these data. Furthermore, the need for new Darwin Core terms was commented upon briefly.

## Introduction

Carabid beetles are model organisms whose systematics, morphology, ecology and evolution have been extensively studied and documented ([Bibr B7539379][Bibr B7539622], Thiele 1977). They respond to biotic and abiotic variations on a regional scale, but also at the ecological landscape and continent level (Vanbergen et al. 2010, Brandmayr et al. 2013). Moreover, Carabidae show several species traits that make them useful as biological indicators (Cajaiba et al. 2018, McGregor and Wahl 2020).

Beech forests are a multi-ecosystems plant cover, widespread all over the Europe, where *Fagussylvatica* is the characterising species of climax stages (Knapp 2011). From the ecogeographic point of view, Knapp (2011) recognises eight units on the basis of three altitudinal belts. From the botanical point of view, a greater variety of beech-dominated plant communities exists in the Atlantic biogeographical region (Piovesan et al. 2011), as mirrored by the NATURA 2000 classification (EU Commission 2013), accounting for twelve different beech forest habitats. The EUNIS classification is even more detailed, accounting, under the 'Beech woodland' category, as many as 160 different types of beech forests.

Beech is one of the most widespread forest tree species in Italy, from the Alps through to the Apennines, down to Sicily, where it reaches its southern distribution limit (Piovesan et al. 2011).

Several research studies on carabids, living in beech forests, have been undertaken in Italy, but data are not directly available in online databases and, in some cases, they have to be "dug out" from hard-copy papers (Brandmayr and Zetto Brandmayr 1984, Brandmayr and Pizzolotto 1988, Chemini and Pizzolotto 1990, Pizzolotto and Brandmayr 1990, Pizzolotto 1993, De Mei et al. 1995, Pizzolotto and Lasen 1997, Pizzolotto and Brandmayr 2014).

## General description

### Purpose

Given the role of carabids as model organisms for environmental monitoring and the pre-eminence of beech ecosystems within the European ecological landscapes, it is very important to provide open access to data on distribution and abundance of these insects.

### Additional information

The study area was a small part of the beech forest ecosystem in the Calabria Region (Italy). In Table 1, the geographic features of the sample sites have been recorded. It is quite problematic to have a unique classification of the vegetation, based on the categories currently applied at European level, because at least four solutions are available according to: i) NATURA 2000 category 9220 (European Commission 2013), subcategory 41.184 of Devillers and Devillers-Terschuren (1996); ii) EUNIS that offers two categories, i.e. category G1.6 "*Fagus* woodland" (Davies et al. 2004; see also https://eunis.eea.europa.eu/habitats.jsp, within which it is possible to find the correct subcategory) and the revised category T17, both corresponding to the same 9220 category of NATURA 2000; and iii) Corine Land Cover category 3.1.1 "Broad-leaved forest" (Bossard et al. 2000), that in Italy can be split down to the 3.1.1.5 "Beech dominated woods" fourth level (ISPRA 2010).

On the basis of the diversity of these classifications, one can argue that, even within Italy, comparisons are difficult amongst, for example, records of the same carabid species living in different regions. On the basis of the sole Darwin Core tag "Habitat", one could provide both the general indication of "beech" and the specific indication of "NAT2k 9220 Apennines beech forests with *Abiesalba*".

This is why new Darwin Core terms should be added, as for example, dwc:habitatNat2k, dwc:habitatEunis, dwc:habitatCLC, to give the possibility to record the vegetation type according to NATURA 2000, EUNIS and Corine Land Cover classifications, respectively.

The dataset records the richness and abundance of carabid species sampled in five sample sites, characterised by the same type of vegetation of beech-dominated forests, over a deep and well-developed forest soil.

Amongst the 28 collected species, only nine were present in all the sample sites. Six of these showed very high annual Activity Density, which are likely to be the characterising species of the studied ecosystem, i.e. *Calathusrotundicollis*, *Calathusfracassi*, *Oreophilusbicolor*, *Nebriakratteri*, *Trechusobtusus* and *Calathusmontivagus*.

The carabids harboured by these ecosystems are of particular importance, not only from a biogeographical point of view, but also with regard to the species traits characterising beetle communities, that showed these to be valuable ecological indicators (Gaublomme et al. 2008, Šerić Jelaska et al. 2011, De Heij and Willenborg 2020).

Unfortunately, it seems that no specific tag is available to include in the dataset the information about species traits, as for example, macropterous vs. brachypterous or about biological features, as for example, spring breeder vs. autumn breeder, predator vs. seed-eater. To help the use of carabids in applied studies, a set of new Darwin Core terms seems to be necessary, as for example, dwc:biolDispersal, dwc:biolPhenology, dwc:biolFeeding, dwc:biolChorology.

## Sampling methods

### Sampling description

Ground beetles were collected, from June to September 1998 (sample sites FaV1-4), and from June to October 2007 (sample site MS4), by pitfall traps, to obtain a “year’s sample”, which is the sum of the collections made during the study. The traps were plastic vessels with an upper diameter of 9.2 cm, a depth of 11 cm and a small opening at 4 cm below the border, to avoid rainwater overflowing, filled with 200 ml of a conservative mixture made of wine vinegar and 5% formaldehyde.

Given the unpredictable events that cause traps to break (e.g. cows, tourists seeking mushrooms), the year sample of each site may be affected by an uneven sampling effort. For this reason, species abundance of a year's sample is evaluated as annual Activity Density (aAD), related to a period of ten days, as follows:

sef = traps * (dd/10)

where sef is the sampling effort evaluated for each collection, traps is the number of active traps, dd is the number of days; then

SEF = Σ sef

gives the total (yearly) sampling effort and

aAD = tot indiv / SEF

gives the annual Activity Density, i.e. the number of individuals per trap, per 10 trapping days, where tot indiv is the total number of individuals captured in that site.

## Geographic coverage

### Description

The study area was in Italy, Calabria, on the Sila Plateau, along the road "Strada delle Vette". Detailed coordinates of the sample sites are provided in the Event Table of the dataset "Carabids in beech forests, Calabria, Italy" (Pizzolotto 2021

### Coordinates

39.272 and 39.333 Latitude; 16.394 and 16.485 Longitude.

## Taxonomic coverage

### Description

Coleoptera, Carabidae: activity density of the sampled species are provided in the Occurrence Table of the dataset "Carabids in beech forests, Calabria, Italy"(Pizzolotto 2021).

## Temporal coverage

### Notes

From June to September 1998 and from June to October 2007

## Usage licence

### Usage licence

Open Data Commons Attribution License

## Data resources

### Data package title

Carabids in beech forests, Calabria, Italy

### Number of data sets

2

### Data set 1.

#### Data set name

event

#### Data format

Darwin Core

#### Number of columns

15

#### Download URL

https://doi.org/10.15468/4sjav7

**Data set 1.** 

Column label	Column description
eventID	Identifier for the set of information associated with the sampling event.
samplingProtocol	Descriptions of the methods or protocols used during sampling.
sampleSizeValue	Number of active pitfall traps.
sampleSizeUnit	One pitfall trap.
samplingEffort	10 * traps/days.
eventDate	The date during which sampling occurred.
startDayOfYear	The earliest integer day of the year on which sampling occurred (e.g. 190 is the 10th of July).
endDayOfYear	The latest integer day of the year on which sampling occurred.
countryCode	The standard code for the country in which the sampling location occurs.
maximumElevationInMetres	The upper limit of the range of elevation in metres.
Habitat	A category or description of the habitat in which the Event occurred.
decimalLatitude	Latitude in decimal degrees.
decimalLongitude	Longitude in decimal degrees.
geodeticDatum	The ellipsoid, geodetic datum or spatial reference system (SRS) upon which the geographic coordinates given in decimalLatitude and decimalLongitude are based.
coordinateUncertaintyInMetres	The horizontal distance (in metres) from the given decimalLatitude and decimalLongitude describing the smallest circle containing the whole of the Location.

### Data set 2.

#### Data set name

occurrence

#### Data format

Darwin Core

#### Number of columns

20

#### Download URL

https://doi.org/10.15468/4sjav7

**Data set 2.** 

Column label	Column description
eventID	Identifier for the set of information associated with sampling event (the same as for the event table).
occurrenceID	An identifier for the Occurrence, possibly a persistent global unique identifier. Here, it is given by the eventID plus the four first letters of genus and species.
basisOfRecord	The specific nature of the data record. Here, all records come from human observation.
individualCount	The number of individuals present at the time of the Occurrence.
organismQuantity	A number or enumeration value for the quantity of organisms. Here, an index of activity density (see next column).
organismQuantityType	The type of quantification system used for the quantity of organisms. Here, the activity density = ind * sampling effort.
LifeStage	The age class or life stage of the Organism(s) at the time the Occurrence was recorded.
occurrenceStatus	A statement about the presence or absence of a Taxon at a Location.
eventDate	The date-time or interval when the occurence was recorded.
scientificName	The full scientific name.
kingdom	Kingdom
phylum	Phylum
class	Class
order	Order
family	Family
genus	Genus
specificEpithet	The name of the first or species epithet of the scientificName.
taxonRank	The taxonomic rank of the most specific name in the scientificName.
Scientific Name Authorship	The authorship information for the scientificName formatted according to the conventions of the applicable nomenclatural Code.
Name Published In Year	The four-digit year in which the scientific Name was published.

## Figures and Tables

**Table 1.** Sample sites geographic features.

	FaV1	FaV2	FaV3	MS4	FaV4
Altitude m a.s.l.	1650	1700	1650	1650	1790
Aspect	S	SE	N	W	N
Inclination	10°	20°	20°	15°	20°
Wood canopy %	90	90	70	95	95
